# Association between hospital volume and outcomes in ovarian cancer: a systematic review

**DOI:** 10.3389/fsurg.2025.1635555

**Published:** 2025-09-11

**Authors:** Qing Luo, Yan Wang, Xiaoyun Zhang

**Affiliations:** Department of Gynaecology and Obstetrics, Jiuquan People’s Hospital, Jiuquan, Gansu, China

**Keywords:** hospital volume, ovarian cancer, surgical outcomes, survival, systematic review

## Abstract

**Background:**

Hospital surgical volume has been proposed as a determinant of ovarian cancer (OC) outcomes, but findings remain inconsistent.

**Objective:**

To systematically assess the association between hospital volume and outcomes in OC patients.

**Methods:**

A systematic search of PubMed, Embase, and Cochrane Library was conducted through January 2025. Fifteen observational studies involving over 100,000 OC patients were included and qualitatively synthesized.

**Results:**

Thirteen of fifteen studies demonstrated that treatment in high-volume hospitals was significantly associated with improved overall survival (OS). Reported benefits included a 5-year OS increase from 22.3% to 55.0%, and up to 3% OS gain per 20 additional surgeries per year. High-volume centers also showed lower perioperative and 90-day mortality (e.g., 0.9% vs. 2.5%), and reduced failure-to-rescue rates. Two studies reported longer progression-free survival (PFS) in high-volume settings. Surgical quality indicators—such as complete cytoreduction and lymphadenectomy—were consistently higher in high-volume hospitals. Despite slightly higher complication rates, these centers had shorter hospital stays and better complication management.

**Conclusions:**

Higher hospital surgical volume is associated with better survival, lower mortality, and superior surgical quality in OC patients. Centralization of OC care may optimize outcomes and should be considered in policy planning.

## Introduction

Ovarian cancer (OC) is one of the most common and lethal cancers affecting women, with an estimated 225,000 new cases and 145,000 deaths annually worldwide (1). Despite improvements in chemotherapy, surgical techniques, and targeted therapies, OC continues to present significant challenges in terms of prognosis, with a 5-year survival rate of approximately 37.4% (2). This poor prognosis is largely attributed to late-stage diagnosis, where effective treatment options are limited (3, 4). Early detection and optimal treatment, particularly complete cytoreductive surgery followed by adjuvant chemotherapy, are critical to improving survival outcomes (5). However, even when similar treatments are administered, patient outcomes can vary considerably, suggesting that factors beyond the treatment regimens may influence clinical results.

One such factor that has been increasingly recognized as a determinant of patient outcomes is hospital volume, defined as the number of OC surgeries performed annually at a given institution (6, 7). A growing body of evidence suggests that hospitals with higher surgical volumes are associated with improved survival rates and lower mortality compared to low-volume centers (8, 9). High-volume hospitals typically have more experienced surgical teams, better access to specialized care, and enhanced perioperative management, all of which contribute to superior patient outcomes (10, 11). Conversely, low-volume hospitals may face challenges such as fewer resources, less specialized expertise, and potentially lower-quality care, which can result in suboptimal outcomes for patients (10, 11). The concept of a volume-outcome relationship is not unique to OC. Numerous studies in other medical disciplines have shown a significant association between higher hospital volume and improved patient outcomes. For instance, a meta-analysis on esophagectomy for cancer found that hospitals with higher surgical volumes were associated with significantly lower postesophagectomy mortality rates (12). Similarly, Tatsuo Hata et al. demonstrated that hospitals performing a greater number of pancreaticoduodenectomy had lower mortality rates, underscoring the importance of hospital volume in determining surgical success (13). In the context of OC, several studies have reported that high-volume hospitals demonstrate superior outcomes, such as higher rates of complete cytoreductive surgery and more timely chemotherapy initiation, both of which are critical for improving survival in OC patients (14, 15). However, the literature on this relationship remains inconsistent. While some studies show a clear survival advantage for patients treated at high-volume centers, others report no significant difference in outcomes (16). These discrepancies may arise from variations in study designs, patient populations, and definitions of hospital volume across different settings. Given the conflicting evidence, a comprehensive and rigorous synthesis of the available data is required. This systematic review seeks to address these gaps by assessing the association between hospital volume and OC outcomes.

The primary objective of this systematic review is to evaluate the impact of hospital volume on OC outcomes. Given the available evidence suggesting that higher hospital volume may be associated with superior outcomes, this review aims to determine whether centralization of OC treatment should be recommended as a standard of care. By synthesizing the current data, we seek to provide clinicians, policymakers, and healthcare systems with evidence-based insights to guide decision-making and improve patient survival.

## Materials and methods

This systematic review was conducted following the Preferred Reporting Items for Systematic Reviews and Meta-Analyses (PRISMA) to ensure methodological rigor and transparency (17). Given the substantial heterogeneity involving hospital volume definitions, study designs, and outcome measures across included studies, a qualitative synthesis was chosen instead of a meta-analysis. We did not register this review on PROSPERO because it was designed as a qualitative synthesis without a pre-specified plan for quantitative pooling considering substantial heterogeneity.

### Search strategy

A comprehensive literature search was conducted using PubMed, Embase, and The Cochrane Library from inception to January 2, 2025 without language limitation. The search strategy was developed using a combination of Medical Subject Headings (MeSH) terms and free-text keywords. The key search domains encompassed OC, hospital volume, and oncologic outcomes, with search terms such as “*ovarian neoplasm” OR “epithelial ovarian cancer”*, “*hospital volume” OR “surgical caseload” OR “high-volume centers”*, and “*overall survival” OR “progression-free survival” OR “postoperative mortality” OR “surgical outcomes”*. Boolean operators (AND, OR) were applied to refine the search, and additional truncation and wildcard symbols were used where appropriate. Additionally, reference lists of included studies and relevant reviews were manually screened for any eligible studies.

### Study selection and data extraction

Observational studies were included if they explicitly investigated the association between hospital volume and OC outcomes. Studies were required to report at least one clinically relevant outcome, including overall survival (OS), progression-free survival (PFS), perioperative mortality, postoperative complications, or hospital length of stay. Studies were excluded if they did not stratify outcomes based on hospital volume, analyzed multiple cancer types without separate subgroup analyses for OC, or were non-original research such as meta-analyses, reviews, case reports, or editorials. Conference abstracts, letters to the editor, and publications without full-text availability were also excluded.

Study selection was performed in two sequential stages by two independent reviewers (LQ and ZXY). First, all retrieved articles underwent title and abstract screening to exclude irrelevant studies. The remaining full-text articles were then assessed against the predefined eligibility criteria. Any discrepancies between the two reviewers were resolved through discussion, and if consensus could not be reached, a third reviewer (WY) was consulted for adjudication. For each eligible study, data were extracted using a standardized data collection form to ensure consistency and minimize bias. Extracted data included study characteristics (first author, year of publication, country, study design, data source, and study period), patient demographics (sample size, median age, stage of disease, and treatment details), and hospital volume definitions (categorization method, quartiles, tertiles, or absolute thresholds). Additionally, information on outcome measures, including OS, PFS, perioperative mortality, postoperative complications, and hospital length of stay, was systematically recorded. Where available, adjustments for confounders (such as patient age, tumor stage, comorbidities, and surgeon experience) were also extracted.

### Quality assessment and risk of bias

The quality and risk of bias for each included study were assessed using the Newcastle–Ottawa Scale (NOS) (18). This tool evaluates observational studies across three domains: selection of study participants, comparability of study groups, and ascertainment of outcomes. Studies were assigned scores based on factors such as representativeness of the cohort, adjustment for confounders, adequacy of follow-up, and the method of outcome measurement. Based on the total NOS score, studies were classified as high quality (≥7 points), moderate quality (4–6 points), or low quality (<4 points). Any discrepancies in scoring between reviewers were resolved through discussion.

### Statistical analysis

Due to substantial heterogeneity including study designs, hospital volume thresholds, and reported outcome measures across included studies, a qualitative synthesis approach was performed instead of a meta-analysis. Studies were summarized based on common patterns in survival outcomes, perioperative mortality, surgical quality indicators, and postoperative complications.

## Results

### Study selection and study characteristics

**Figure 1:**
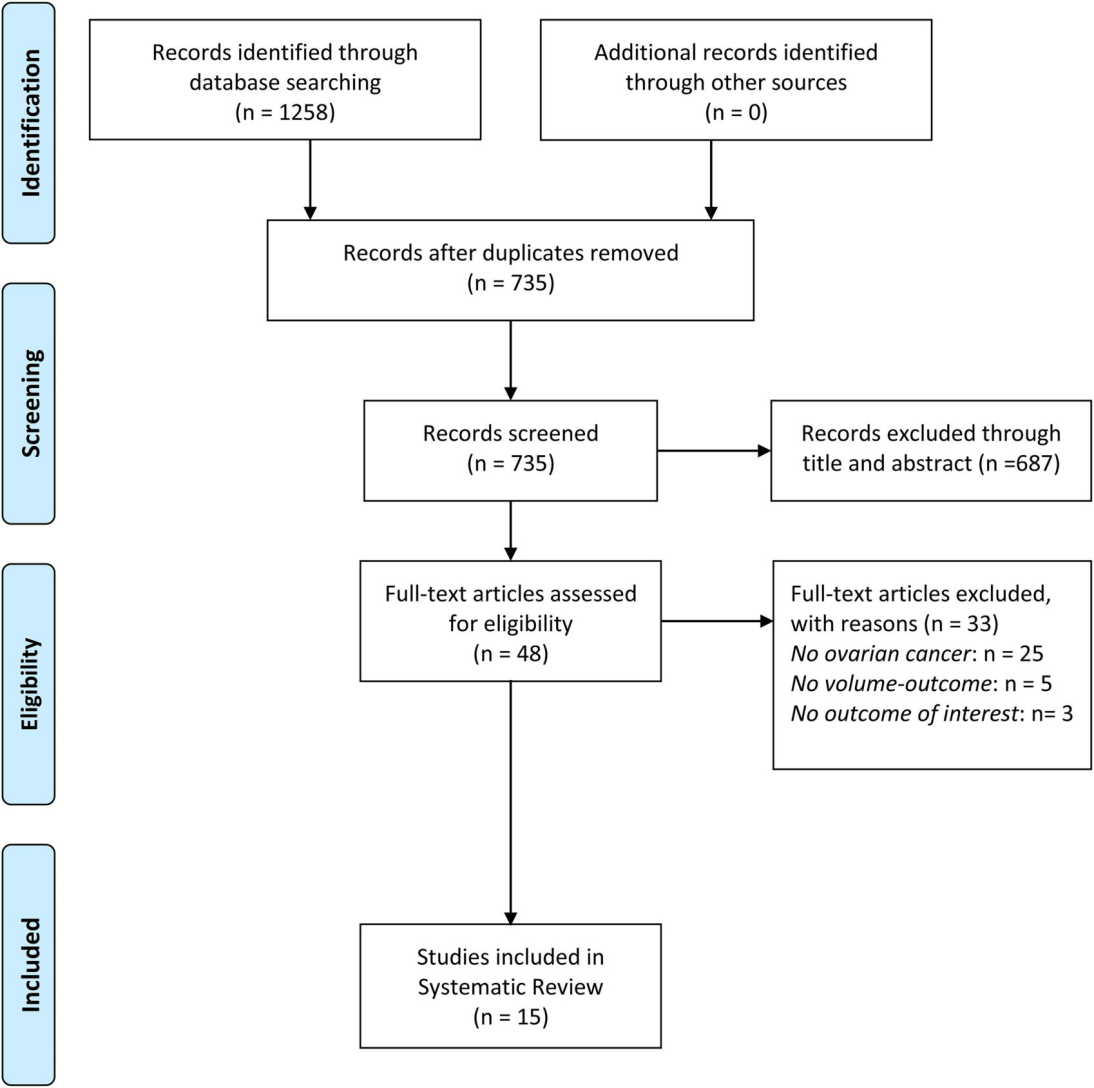
The PRISMA flow chart of study selection.

The initial literature search identified 1,258 articles. After screening titles and abstracts, 48 studies were retrieved for full-text review. Ultimately, a total of 15 observational studies were included in the systematic review with sample sizes ranging from 231 to 104,766 patients (14–16, 19–30). Figure 1 illustrates the study selection process. These studies were conducted in diverse healthcare systems, including the United States, Japan, Finland, France, Belgium, and Brazil. Many studies relied on nationwide cancer registries, such as the Surveillance, Epidemiology, and End Results-Medicare database, the National Cancer Database, Belgian Cancer Registry, and the Finnish nationwide study. Hospital volume was categorized using different classification methods. Some studies divided hospitals into quartiles based on the number of OC surgeries performed annually, while others used absolute numerical thresholds, defining high-volume hospitals as those performing ≥20 cases per year. A few studies adopted tertile-based classifications, whereas others analyzed volume as a continuous variable, using statistical cutoffs to define high- and low-volume categories. The NOS score was used to assess the quality of the studies. Out of the 15 studies, 13 studies were classified as high quality, and 2 studies were rated as moderate quality. The main reasons for moderate quality in cohort studies included selection bias, particularly in how hospital volume was assigned, and attrition bias in studies with high dropout rates (especially in long-term follow-up). The detailed characteristics and outcomes of included studies were showed in Tables 1, 2 and Supplementary Table S1.

**Table 1:** Baseline characteristics of included studies.

Author (Year)	Data source	Study period	Study center	Country	No. of patients	Hospital volume groups (cases/year)	Outcomes of interest	Adjusted confounders
Ioka et al. 2004 (22)	Osaka Cancer Registry's database	1975–1995	Population-based	Japan	3,523	High: >8.8 Medium: 4.0 Low: 2.0 Very low: <1	5-year survival	Age, histologic type, cancer stage and hospital procedure volume
Schrag et al. 2006 (16)	SEER-Medicare linked database	1992−1999	Population-based	United States	2,952	High: 29–93 Intermediate: 13–28; Low: 1–12	60-day and 2-year postoperative mortality, Overall survival,and Length of hospital stay	Age, race, AJCC stage at diagnosis, comorbidity, socioeconomic status, and population density
Kumpulainen et al. 2009 (24)	Finnish Cancer Registry	1999	Multi-center	Finland	275	High: >21; Intermediate: 11−20; Low: 0–10	5-year cancer-specific survival Median disease-free survival	Age-group, FIGO stage, hospital volume, operating physician, residual disease, degree of differentiation and primary chemotherapy
Mercado et al. 2010 (25)	Cancer registries (California, Washington, New York, Florida)	1991–2004	Population-based	United States	31,897	Low: 0–4 Middle: 5–9 High: 10–19 Very high: >20	Overall survival	Age, comorbidity, and hospital location
Bristow et al. 2010 (19)	National Cancer Data Base	1996–2005	Multi-center	United States	45,929	Low: 0–9 Intermediate: 9–20 High: 21–35 Very high: >35	Overall survival	Treatment paradigm, pathologic stage, ethnicity, age, payer status, household income, and tumor grade
Wright et al. 2012 (30)	Nationwide Inpatient Sample	1988–2009	Population-based	United States	36,624	No available	Mortality rate. Major complication rate. Failure to rescue rate	Age, year of surgery, race, comorbidity, urgency of operation, performance of extended cytoreduction, and hospital teaching status
Seagle et al. 2017 (27)	National Cancer Database	1998–2011	Population-based	United States	104,766	No available	Overall survival	Age categories, stage, grade, and histology
Wright et al. 2017 (29)	National Cancer Database	2004–2013	Population-based	United States	100,725	Very low: 0–2 Low: 2.01–5 Middle: 5.01–9 High: 9.01–19.9 Very high: >20	2-year overall survival; 5-year overall survival	Age, race, insurance status, education, location, comorbidity, year of diagnosis, stage, grade, histology, and lymph node dissection
Spencer et al. 2017 (15)	National Cancer Database	2004–2012	Population-based	United States	24,827	Low: 0–10 Intermediate: 11–20 High: 21–30 Ultra-high: >31	30-day mortality 90-day mortality	Age, race–ethnicity, income, Charlson comorbidity index, insurance status, hospital volume, distance from place of residence to the hospital, receipt of neoadjuvant chemotherapy, and year of diagnosis
Huguet et al. 2018 (21)	Prospectively implemented regional databases	2012	Multi-center	France	231	Low: <12 High: >12	Progression-free survival	Age, histology, FIGO stage, grade, cancer history, neoadjuvant chemotherapy, and the presence of ascites
Wright et al. 2019 (28)	New York Statewide Planning and Research Cooperative System database	2000–2014	Multi-center	United States	25,044	No available	Perioperative mortality; Perioperative morbidity; hospital length of stay	Surgeon and hospital volume quartiles, cancer, cytoreduction, age, year, race/ethnicity, insurance status, admission type, comorbidity, and procedure score
Knisely et al. 2020 (23)	National Cancer Database	2004–2016	Population-based	United States	56,834	No available	30-day mortality, 90-day mortality, 5-year overall survival	Tumor characteristics (histology, grade), and chemotherapy
Moterani et al. 2020 (26)	Fundação Oncocentro de São Paulo cancer registry	2000–2018	Population-based	Brazil	6,111	High: >20 Low: <20	10-year overall survival	Age, tumor summary stage, tumor histology, chemotherapy (yes or no), hospital volume (high or low), and hospital class (teaching or community
Frost et al. 2022 (20)	National Cancer Database	2004–2016	Population-based	United States	4,640	No available	10-year overall survival	Patient, clinical, and hospital factors
Savoye et al. 2023 (14)	Belgian Cancer Registry	2014–2018	Population-based	Belgium	3,988	Low: 0–14 Intermediate: 15–28 High: 29–49 Ultra-high: >50	5-year overall survival 30-day post-operative mortality	Age at diagnosis, anatomic site, WHO performance status, diabetes, cardiovascular comorbidity, respiratory comorbidity, inpatient bed days during year prior to incidence date, tumour stage, histological type

**Table 2:** Methodological quality assessment of included studies by Newcastle–Ottawa scales.

Study	Selection				Comparability	Outcome			Total score
	Exposed cohort	Nonexposed cohort	Ascertainment of exposure	Outcome of interest		Assessment of outcome	Length of follow-up	Adequacy of follow-up	
Ioka 2004 (22)	*	*	*	*	**	*	*	——	8
Schrag et al. 2006 (16)	*	*	*	*	**	*	*	——	8
Kumpulainen et al. 2009 (24)	*	*	*	*	**	*	*	——	8
Mercado et al. 2010 (25)	*	*	*	*	**	*	*	——	8
Bristow et al. 2010 (19)	*	*	*	*	*	*	*	——	7
Wright et al. 2012 (30)	*	*	*	*	*	*	——	——	6
Seagle et al. 2017 (27)	*	*	*	*	*	*	——	——	6
Wright et al. 2017 (29)	*	*	*	*	*	*	*	——	7
Spencer et al. 2017 (15)	*	*	*	*	**	*	*	——	8
Huguet et al. 2018 (21)	*	*	*	*	**	*	*	——	8
Wright et al. 2019 (28)	*	*	*	*	**	*	——	——	7
Knisely et al. 2020 (23)	*	*	*	*	**	*	*	——	9
Moterani et al. 2020 (26)	*	*	*	*	*	*	*	——	7
Frost et al. 2022 (20)	*	*	*	*	**	*	*	——	8
Savoye et al. 2023 (14)	*	*	*	*	*	*	*	——	7

### Association between hospital volume and outcomes

#### Overall survival (OS)

A total of eight studies explored the relationship between hospital volume and OS in OC. Of these, seven studies revealed a significant survival benefit for patients treated in high-volume hospitals. A population-based study in Osaka, Japan, revealed that 5-year relative survival for ovarian cancer was 55.0% in high-volume hospitals vs. 22.3% in very-low-volume hospitals. After adjusting for age, stage, and histology, patients in very-low-volume centers had a 60% higher mortality risk (HR = 1.6) (22). In a multi-state cohort of 31,897 patients with stage IIIC/IV OC, treatment at higher-volume hospitals was associated with significantly better survival: high-volume (HR = 0.89, 95% CI: 0.86–0.93) and very-high-volume hospitals (HR = 0.79, 95% CI: 0.76–0.83), compared to low-volume centers (25). In a national cohort of 45,929 stage IIIC/IV OC patients, hospital surgical volume was independently linked to OS and a threshold of ≥21 cases/year may be critical to improving survival (19). In a national cohort of 104,766 OC patients from the NCDB, higher hospital volume was significantly associated with improved overall survival. Each 20-patient/year increase in mean annual hospital volume reduced the hazard of death by 3% (HR = 0.97; 95% CI: 0.96–0.99; *P* < .001). Among women with stage III–IV high-grade serous OC, mean OS increased from 49.4 to 54.7 months when treated at hospitals with 5 to 50 cases per year (27). In a national cohort of 100,725 OC patients, hospital volume was significantly associated with overall survival. Adjusted 2-year survival increased from 64.4% at low-volume hospitals (≤2 cases/year) to 77.4% at high-volume centers (≥20 cases/year), and 5-year survival rose from 39.3% to 51.0% (*P* < .001 for both) (29). In a cohort of 4,640 women with stage II–IV serous OC, hospital case volume was not significantly associated with 10-year survival in the overall population (OR = 1.15, 95% CI: 0.92–1.44). However, among women who survived ≥5 years from diagnosis, treatment at high-volume hospitals (≥20 cases/year) was associated with increased odds of 10-year survival (OR = 1.33, 95% CI: 1.02–1.74) (20). In a Belgian population-based cohort of 3,988 women with invasive epithelial OC, hospital volume was significantly associated with survival (14). Compared to the highest-volume centers (≥50 patients/5 years), patients treated in the lowest-volume quartile (1–14 patients/5 years) had a 47% higher risk of death within 5 years (adjusted HR = 1.47; 95% CI: 1.11–1.93; *P* = .006). Median survival was 4.2 vs. 1.7 years. Each additional patient treated annually reduced the mortality hazard by 1.1%, up to a threshold of 9 patients/year. Only one study showed no significant correlation between hospital volume and OS in OC. A SEER-Medicare study by Schrag et al. found that hospital volume is not strong predictors of survival outcomes following surgery for OC among women aged 65 years or older (16). Among all the included studies involving OS, most of them were high-quality, with effect estimates remaining robust after multivariable adjustment. The higher-quality evidence lends substantial weight to the conclusion that institutional surgical volume is a strong and independent predictor of survival outcomes in OC.

#### Progression-Free survival (PFS) and disease-free survival (DFS)

Only two studies investigated the relationship between hospital volume and PFS or DFS in OC. In a prospective nationwide study of 275 patients with epithelial OC in Finland (24), higher hospital operative volume was significantly associated with improved DFS. Median DFS was 33 months, and in multivariate analysis, increasing hospital volume was associated with longer DFS. When categorized, patients treated in high-volume hospitals (>20 cases/year) had higher DFS rates than those in lower-volume centers. Similarly, in a population-based cohort of 231 patients with epithelial OC in France, treatment at high-volume hospitals was significantly associated with longer PFS (21). Median PFS was 20.0 months in high-volume hospitals vs. 14.2 months in low-volume hospitals. After adjustment using multivariate analysis and inverse probability weighting, the hazard of progression or death was nearly halved in high-volume centers, highlighting the strong benefit of centralization in first-line treatment. To sum up, PFS or DFS data were reported in two high-quality studies and all of them demonstrated a statistically significant advantage for patients managed in high-volume centers. The predominance of positive findings suggests that hospital volume exerts a meaningful influence on disease control, likely through improved surgical cytoreduction rates and adherence to evidence-based adjuvant therapy protocols.

#### Perioperative mortality

A total of ten studies investigated the relationship between hospital volume and mortality in OC. In a SEER–Medicare cohort of 2,952 women aged ≥65 with OC, hospital volume was modestly associated with 2-year mortality: 45.2% at low-volume hospitals, 41.1% at intermediate-, and 40.4% at high-volume centers (16). No significant differences were observed in 60-day mortality across volume categories. In a nationwide Finnish cohort of 275 epithelial OC patients, higher hospital operative volume was not associated with improved 5-year cancer-specific mortality in full multivariate analysis (HR = 0.998; 95% CI: 0.981–1.016; *P* = 0.857) (24). In a cohort of 36,624 OC patients undergoing surgery, inpatient mortality decreased with hospital volume: 1.8% at low-volume hospitals, 1.6% at intermediate-, and 1.5% at high-volume centers (*P* < .001) (30). Although complication rates were higher in high-volume hospitals (24.6% vs. 20.4%), the failure-to-rescue rate—mortality after a major complication—was nearly halved (4.9% vs. 8.0%). After adjustment, patients at low-volume hospitals were 48% more likely to die after a complication (OR = 1.48; 95% CI: 1.11–1.99), underscoring that lower mortality in high-volume centers is driven by superior management of complications. In a national NCDB cohort of 104,766 ovarian cancer patients, each 20-patient/year increase in mean annual hospital volume reduced the hazard of death by 3% (HR = 0.97; 95% CI: 0.96–0.99; *P* < .001), indicating a strong volume–mortality gradient (27). In a cohort of 24,827 patients with high-grade serous OC, patients at high-volume hospitals were associated with lower 90-day mortality (adjusted OR = 0.60; 95% CI: 0.38–0.96; *P* = .034) compared to low-volume centers (15). Unadjusted 90-day mortality declined from 5.66% to 3.37% with increasing hospital volume, supporting 90-day mortality as a meaningful quality metric. In a population-based cohort of 231 epithelial OC patients in France, treatment at high-volume hospitals (≥12 cases/year) was associated with significantly lower risk of death (21). Using inverse probability weighting, patients in low-volume hospitals had a 1.94-fold higher hazard of death compared to those in high-volume centers. In a statewide cohort of 25,044 OC patients, perioperative mortality declined with increasing hospital volume, from 2.5% in the lowest quartile to 0.9% in the highest. After adjustment, high-volume hospitals were associated with a 33% lower mortality risk compared to low-volume centers (RR = 0.67; 95% CI: 0.46–0.97), highlighting the survival benefit of care centralization despite similar complication rates across hospitals (28). In a cohort of 56,834 women with stage II–IV OC, treatment at high-volume hospitals was associated with slightly lower 90-day mortality (6.7% vs. 7.5%), though the adjusted risk reduction was not significant (aRR = 0.95; 95% CI: 0.71–1.27), suggesting limited volume-related benefit on short-term mortality (26). In a nationwide cohort of 3,988 OC patients in Belgium, hospital volume was significantly associated with mortality. Patients treated in the lowest-volume hospitals (1–14 cases/5 years) had a 47% higher risk of death within five years compared to those in the highest-volume centers (≥50 cases/5 years) (adjusted HR = 1.47; 95% CI: 1.11–1.93; *P* = .006). Thirty-day postoperative mortality was also significantly higher in the lowest surgical volume quartile (8.6%) than in the highest (1.3%), with adjusted OR = 4.78 (95% CI: 2.04–11.19; *P* = .0003) (14). Collectively, while moderate heterogeneity exists in effect sizes and statistical significance, the weight of high-quality evidence indicates that higher hospital volume is associated with reduced perioperative mortality in OC, primarily through improved management of complications rather than reduced event rates.

#### Surgical quality indicators: R0 resection and lymphadenectomy

Surgical quality is a major determinant of long-term survival in OC. A consistent association has been observed between hospital surgical volume and the likelihood of achieving complete cytoreduction (R0 resection) and performing lymphadenectomy in OC patients. In a nationwide Finnish cohort of 275 women, Kumpulainen et al. found that treatment at higher-volume hospitals significantly increased the likelihood of no macroscopic residual disease, with a 20% increase in odds per 10 additional cases annually (24). Similarly, Wright et al. analyzed 36,624 cases in the U.S. and reported that high-volume centers were more likely to perform extended cytoreductive procedures and lymphadenectomy (30). In another study using SPARCS data, Wright et al. showed that high-volume hospitals conducted significantly more complex cytoreductive surgeries, including rectosigmoid resection, diaphragm resection, and splenectomy, reflecting greater surgical aggressiveness and completeness (28). Collectively, these findings support the notion that hospital volume contributes to surgical quality through increased rates of complete resection and adherence to staging procedures.

#### Postoperative complications and length of hospital stay

Postoperative complications were consistently associated with prolonged hospital stays among OC patients. Only three studies reported postoperative complications and length of hospital stay (16, 28, 30). Wright et al. reported that although high-volume hospitals had higher complication rates, the proportion of patients with extended hospitalizations were significantly lower, reflecting more efficient complication management (28, 30). Similarly, Schrag et al. found that patients treated in high-volume hospitals had shorter mean LOS (3.5 vs. 5.5 days; *P* < .001), even after adjusting for patient and disease characteristics (16). The available evidence supports the premise that institutional volume correlates with more effective perioperative complication prevention and management.

## Discussion

This systematic review indicates that higher hospital surgical volume is generally associated with better outcomes for OC patients. Most studies found that treatment at high-volume centers correlates with improved OS, as well as lower perioperative mortality and morbidity.

### Comparison with previous literature

The findings of this review align with previous studies investigating the volume-outcome relationship in other cancers. For example, in liver cancer, pancreatic cancer, and gastric cancer, high-volume hospitals have been shown to consistently achieve better outcomes, including lower mortality, reduced complications, and improved survival (31–33). Similar patterns were observed in OC, where hospitals with greater experience and surgical volume demonstrated superior surgical outcomes and improved survival rates. In OC specifically, several individual studies have suggested that high-volume centers provide better survival outcomes. However, these studies often lacked the statistical power or robust methodologies necessary to draw definitive conclusions. Our systematic review resolves these inconsistencies by synthesizing data from a substantial number of studies, providing stronger evidence for the impact of hospital volume on OC outcomes.

### Potential mechanisms

The significant association between hospital volume and OC outcomes likely stems from several interconnected mechanisms related to experience, specialization, and resource availability. One of the most prominent mechanisms underlying the volume-outcome relationship in OC is the surgical expertise provided by high-volume centers (34). The complexity of OC surgery—particularly cytoreductive surgery aimed at achieving complete cytoreduction—requires high levels of technical skill, precision, and experience. High-volume hospitals, which perform a greater number of surgeries annually, allow surgeons to develop and refine these skills, leading to fewer complications and better outcomes. Additionally, high-volume hospitals often have multidisciplinary teams that work collaboratively to develop comprehensive treatment plans for OC patients (35). These teams typically consist of specialists in surgery, oncology, pathology, radiology, and palliative care, which allows for a holistic approach to patient care. By involving experts from multiple fields, high-volume hospitals are able to provide personalized treatment plans that are tailored to the specific needs of each patient. Finally, high-volume hospitals are often more likely to have standardized treatment protocols and quality assurance measures in place (36). Standardization ensures that all patients receive evidence-based care, regardless of individual clinician preferences or hospital shifts. Protocols that emphasize timely chemotherapy initiation, appropriate surgical techniques, and postoperative monitoring can significantly improve patient outcomes by reducing variability in care delivery.

### Clinical implications

The results of this study have significant implications for clinical practice and healthcare policy. If hospital volume is indeed a critical determinant of survival and mortality in OC, it underscores the need to centralize care in high-volume centers. Centralizing OC care could not only improve patient outcomes but also optimize the use of specialized resources. High-volume hospitals are more likely to have multidisciplinary teams and advanced technologies that contribute to better surgical outcomes and more timely treatment. In addition to volume, the surgical approach and extent of cytoreduction are critical determinants of outcomes in OC. High-volume centers often adhere to an aggressive surgical philosophy aimed at achieving complete cytoreduction (R0), which is strongly linked to improved survival. Recent data support this relationship: for example, Aksan et al. showed that ultra-radical cytoreductive surgery (vs. standard surgery) can improve progression-free survival and may enhance overall survival in advanced-stage disease (37). This suggests that the volume–outcome benefit is partly mediated by higher surgical radicality and expertise at high-volume hospitals, where teams pursue maximal tumor resection. We now emphasize that hospital volume alone is not sufficient—the ability to achieve R0 resection, reflecting surgical skill and effort, is a key factor underlying better outcomes in high-volume settings. These findings also suggest that patients in low-volume centers may benefit from referral to high-volume centers, particularly for advanced-stage disease where treatment is more complex and outcomes are more dependent on surgical expertise. This referral model could potentially bridge the gap in outcome disparities between low- and high-volume centers, ensuring that all patients receive the highest standard of care available.

### Limitations

This review has several limitations that must be acknowledged. Firstly, all included studies were observational in design (mostly retrospective cohorts), so the findings are associations and cannot prove causation owing to potential potential bias. Although most studies attempted multivariable adjustments, residual confounding is possible. Secondly, an important limitation of the current evidence base is the substantial heterogeneity in how hospital surgical volume is defined across studies, with reported thresholds ranging from as few as eight to more than fifty OC cases per year. Such variability inevitably constrains the comparability of outcomes and may dilute or obscure the true magnitude of the volume–outcome association. Although we sought to improve interpretability in the present review by harmonising reported thresholds into standardised low-, intermediate-, and high-volume categories where possible, this approach cannot fully overcome the methodological inconsistencies inherent in the source literature. The development and adoption of consensus-based, evidence-informed cut-offs—ideally stratified by disease type, healthcare system, and resource setting—would facilitate more robust cross-study comparisons, enable high-quality meta-analysis, and support the establishment of international benchmarks for volume-based quality indicators in ovarian cancer care. Thirdly, there is also the possibility of publication bias—studies showing a positive volume–outcome relationship may have been more likely to be published in high-impact journals, whereas analyses finding no significant difference might be underreported. We attempted to be comprehensive in our literature search, but it is possible that some negative or small studies were missed, or that non-English publications were not fully captured, which could skew the review's perspective. Finally, our focus on hospital volume precluded analysis of surgeon-specific volume or individual provider skill. The experience of the operating surgeon is a known contributor to OC outcomes, and high-volume centers often concentrate high-volume surgeons. We acknowledge that not adjusting for surgeon volume in our included studies may confound the observed hospital-level effects. In other words, part of the survival benefit at high-volume hospitals could derive from the expertise of their surgeons. This limitation has been noted, as it underscores the need for caution when attributing improved outcomes solely to the hospital volume.

## Conclusions

This systematic review highlights a clear association between higher hospital volume and improved outcomes in OC patients. Centralizing care at high-volume institutions appears essential for achieving optimal surgical and survival outcomes. However, effective policy implementation requires careful consideration of equity and accessibility. Ongoing research should focus on standardizing volume definitions, controlling confounding variables rigorously, and exploring broader international comparisons to guide evidence-based healthcare policy development.

## Data Availability

The original contributions presented in the study are included in the article/Supplementary Material, further inquiries can be directed to the corresponding author.
